# Recent Advances in Biomarkers of Systemic Sclerosis-Associated Interstitial Lung Disease: Clinical Relevance and Therapeutic Perspectives

**DOI:** 10.3390/life16091420

**Published:** 2026-08-27

**Authors:** Rasha-Ioana Rămoiu-Shehada, Anca Emanuela Mușetescu, Lucian-Mihai Florescu, Alesandra Florescu, Paulina-Lucia Ciurea

**Affiliations:** 1 Doctoral School, University of Medicine and Pharmacy of Craiova, 200349 Craiova, Romania; rasha.ramoiu@umfcv.ro; 2 Department of Rheumatology, University of Medicine and Pharmacy of Craiova, 200349 Craiova, Romania; anca.musetescu@umfcv.ro (A.E.M.); paulina.ciurea@umfcv.ro (P.-L.C.); 3 Department of Radiology and Medical Imaging, University of Medicine and Pharmacy of Craiova, 200349 Craiova, Romania; lucian.florescu@umfcv.ro

**Keywords:** systemic sclerosis, interstitial lung disease, biomarkers, pulmonary fibrosis

## Abstract

Systemic sclerosis-associated interstitial lung disease (SSc-ILD) is the leading cause of mortality in patients with systemic sclerosis and a major clinical challenge due to its heterogeneous presentation and variable disease course. Reliable biomarkers may improve disease assessment, risk stratification, and therapeutic decision-making. This narrative review summarizes current evidence on established and emerging serum and molecular biomarkers in SSc-ILD and their potential clinical applications. Recent advances have shifted the focus from single-marker assessment toward integrated biomarker profiling, reflecting the complex pathogenesis of pulmonary fibrosis. KL-6 and surfactant protein D (SP-D) have been associated with pulmonary involvement and radiographic features of SSc-ILD. Inflammatory mediators, including interleukin-6 (IL-6), and autoantibodies such as anti-topoisomerase I (anti-Scl-70) have been associated with disease severity and an increased risk of pulmonary progression. Emerging biomarkers related to epithelial injury, extracellular matrix remodelling, and vascular dysfunction may provide additional insights into the biological mechanisms underlying SSc-ILD. Current evidence highlights the potential of circulating and molecular biomarkers to refine risk stratification and prognostic assessment in SSc-ILD. Prospective validation and methodological standardization remain necessary to determine their clinical utility and potential integration into personalized disease management.

## 1. Introduction

Systemic sclerosis (SSc) represents a chronic, autoimmune condition defined by fibrosis of the skin and multiple internal organs and blood vessel injury [1]. Immune system dysregulation underlies these pathological processes, sustaining prolonged inflammation and driving the overproduction and accumulation of collagen along with other extracellular matrix components [2]. Based on the extent of skin involvement, SSc is commonly classified into limited cutaneous systemic sclerosis (lcSSc) and diffuse cutaneous systemic sclerosis (dcSSc), which are associated with distinct patterns of organ involvement and disease course. SSc-specific autoantibodies are important serological markers with established roles in disease diagnosis, classification, and clinical phenotyping. Among these, anticentromere antibodies (ACA), anti-topoisomerase I antibodies (ATA; anti-Scl-70), and anti-RNA polymerase III antibodies (anti-RNAP III) are included in the 2013 American College of Rheumatology/European Alliance of Associations for Rheumatology (ACR/EULAR) classification criteria for SSc [3].

Epidemiological studies indicate that male patients with SSc more frequently display a severe disease phenotype, characterised by diffuse cutaneous involvement, greater exposure to smoking and occupational risk factors, and higher prevalence of ATA and anti-U3 RNP antibodies. By contrast, female patients are more commonly associated with limited cutaneous disease, overlap with systemic lupus erythematosus, and ACA positivity [4]. These sex-related differences are clinically relevant, with male patients showing a higher frequency and greater severity of interstitial lung and cardiac involvement, which may contribute to poorer survival, whereas pulmonary arterial hypertension (PAH) represents a more prominent cause of mortality among female patients [5]. Several clinical and serological variables have been associated with the development and progression of ILD in patients with SSc. Among the most consistently reported risk factors are dcSSc, African American ancestry, older age at disease onset, shorter disease duration at the time of evaluation, and seropositivity for ATA antibodies. Collectively, these factors are associated with an increased risk of pulmonary involvement and may identify patients more likely to develop a progressive course of SSc-ILD [6,7].

Pulmonary involvement is widely recognised as a major contributor to morbidity and mortality in SSc, with ILD and PAH representing key factors associated with adverse clinical outcomes [5,8]. While advances in management have mitigated the mortality associated with renal and peripheral vascular complications in SSc, SSc-ILD remains a primary driver of disease-related mortality, frequently associated with progressive functional decline and unfavourable clinical outcomes [9]. Evidence from post mortem investigations suggests that moderate-to-severe pulmonary fibrotic changes are present in approximately 75% of patients with SSc, whereas clinically apparent restrictive ventilatory dysfunction is reported in only about one-third of cases [10,11]. Histopathological examination of lung biopsy specimens from patients with SSC-ILD predominantly reveals a nonspecific interstitial pneumonia (NSIP) pattern, reported in approximately 78% of cases. Usual interstitial pneumonia (UIP), which is more typically associated with idiopathic pulmonary fibrosis (IPF), is less frequently observed in SSc-ILD, with reported frequencies ranging from 10% to 15% [12].

Evidence from large, ethnically diverse multicentre cohorts indicates that African American patients with SSc may exhibit a distinct clinical and immunological phenotype, characterised by earlier disease onset, a higher frequency of diffuse cutaneous involvement (51–57%), and a greater burden of pulmonary fibrosis compared with Caucasian and Hispanic populations [13]. Data from the GENISOS cohort showed that clinically significant pulmonary involvement may develop early in the disease course, affecting approximately 25% of patients within the first three years following diagnosis. Diffuse cutaneous disease was present in more than half of the cohort, while African American patients showed a higher prevalence of pulmonary fibrosis [14]. Findings from the Pittsburgh Scleroderma Databank and the GRASP cohort further identified a serological profile characterised by higher frequencies of ATA antibodies (27–30%), anti-U3 RNP antibodies (16–40%), and anti-U1 RNP antibodies (16–18%), together with a relatively low prevalence of ACA antibodies. African ancestry was also identified as an independent predictor of severe ILD and progressive pulmonary fibrosis, even within comparable autoantibody subsets, suggesting that ancestry-related biological factors may contribute to differences in pulmonary disease severity and outcomes in SSc [15].

High-resolution computed tomography (HRCT) is considered the gold standard for the detection of ILD in patients with SSc, providing detailed characterization of pulmonary parenchymal abnormalities and facilitating the detection of early interstitial changes [16,17]. The diagnostic assessment of SSc-ILD may be further supported by the ILD-RISC score, a validated multiparametric tool integrating clinical, serological, and functional variables to identify patients at increased risk of ILD. By facilitating risk stratification and guiding the selective use of HRCT, the ILD-RISC score may contribute to earlier detection while potentially reducing unnecessary imaging [18]. Recent multicentre evidence indicates that ILD can also develop during later stages of SSc, beyond the early disease period traditionally associated with the highest risk. Although late-onset ILD occurs less frequently, its clinical severity and progression were comparable to those observed in earlier-onset disease, with nearly half of affected patients developing progressive lung involvement. These findings highlight that the risk of clinically significant ILD extends beyond the early disease course and support continued pulmonary surveillance in the patients with SSc [19]. Across published cohorts, the prevalence of ILD detected by HRCT varies considerably, ranging from approximately 22% to 84% with NSIP being more frequently reported than UIP [20].

The prognostic relevance of pulmonary involvement in SSc is further supported by mortality data from the European Alliance of Associations for Rheumatology (EULAR) Scleroderma Trials and Research (EUSTAR) cohort, which prospectively followed 5860 patients with SSc. A total of 284 deaths were recorded, with detailed information on the cause of death available for 234 cases. More than half of the reported deaths (55%) were directly attributed to SSc, whereas 41% were related to non-SSc causes and the remaining 4% could not be classified. Among SSc-related deaths, pulmonary fibrosis accounted for the largest proportion (35%), followed by PAH and cardiac complications, each accounting 26%. Pulmonary restriction and reduced diffusing capacity of the lung were also identified as independent risk factors for mortality. These findings underscore the prognostic relevance of pulmonary involvement in SSc and support the importance of early identification and longitudinal assessment of patients at risk of progressive lung disease [21,22].

## 2. Research Methods

This narrative review was based on a targeted literature search of PubMed/MEDLINE, Scopus, and Web of Science to identify relevant studies on biomarkers in SSc-ILD published between March 1969 and April 2026. Earlier seminal studies were also considered when relevant to the biological or mechanistic context of specific biomarkers.

The search strategy combined terms related to systemic sclerosis, interstitial lung disease, and biomarkers, including “systemic sclerosis”, “scleroderma”, “SSc-ILD”, “interstitial lung disease”, “biomarker”, “serum biomarker”, “circulating biomarker”, “molecular biomarker”, “autoantibodies”, “cytokines”, “chemokines”, “epithelial injury”, “endothelial dysfunction”, “immune activation”, “extracellular matrix remodelling”, and “fibrosis”. Searches were further refined using the names of individual biomarkers and biomarker families, with Boolean operators (AND and OR) applied as appropriate. Reference lists of key original studies and relevant reviews were manually screened to identify additional publications.

Studies were selected based on their relevance to the objectives of the review, with priority given to original research investigating circulating, serological, and other molecular biomarkers in SSc-ILD. Studies evaluating associations between candidate biomarkers and disease presence, severity, progression, prognosis, or therapeutic response were considered. Prospective and retrospective cohorts, case–control and cross-sectional studies, and clinical trials were included, while systematic reviews and meta-analyses were consulted for contextualization and identification of additional primary studies. Case reports, editorials, commentaries, and publications with insufficient relevant data were excluded from the core evidence synthesis.

Given the narrative design of the review, study selection was guided by relevance, scientific quality, and contribution to the understanding of biomarker biology and clinical utility in SSc-ILD. Biomarkers were synthesized according to their biological and pathophysiological relevance and discussed in relation to their potential diagnostic, prognostic, predictive, and monitoring value, with particular emphasis on emerging biomarkers and multimarker approaches.

## 3. Pathogenesis

Systemic sclerosis (SSc) is a heterogeneous autoimmune connective tissue disorder characterised by a complex and incompletely elucidated pathogenesis, involving a dynamic interplay between genetic predisposition, environmental exposures, and immune-mediated mechanisms. Current evidence supports a multifactorial model in which genetic, epigenetic, and environmental influences contribute to vascular injury, immune dysregulation, and progressive fibrosis, which are closely interconnected and mutually reinforcing processes. In SSc-ILD, the interplay between these pathogenic mechanisms, particularly immune dysregulation and persistent fibroblast activation, contributes to the development and progression of pulmonary fibrosis [23]. Genetic susceptibility is supported by association with multiple loci, many of which overlap with those implicated in other autoimmune diseases, including HLA class II alleles, as well as PTPN22 [24], STAT4 [25,26], IRF5 [27], and NLRP1. This genetic framework is further modulated by epigenetic mechanisms, particularly non-coding RNAs such as miR-21, miR-29, and miR-155, which have been implicated in the regulation of profibrotic transcriptional programs, extracellular matrix (ECM) turnover, and fibroblast activation. Additional regulatory mechanisms, including chromatin remodelling and bromodomain-mediated transcriptional control, may also contribute to persistent fibrogenesis [28]. Although genetic and epigenetic mechanisms have been implicated in SSc pathogenesis, their specific contribution to the development and progression of SSc-ILD remains incompletely understood.

Environmental and infectious exposures have been investigated as potential disease triggers in genetically susceptible individuals. Occupational exposure to crystalline silica and organic solvents such as trichloroethylene, has been associated with increased risk of SSc. Infectious agents, including cytomegalovirus, Epstein–Barr virus, and parvovirus B19—have also been proposed as potential contributors to disease development; however, the available evidence remains insufficient to establish a causal relationship between these infections and SSc [29,30].

Early abnormalities in SSc occur at the level of microvasculature and are characterised by endothelial cell injury and dysfunction. Endothelial activation results in increased expression of adhesion molecules including ICAM-1 and VCAM-1, enhanced chemokine and cytokine release, and recruitment of inflammatory cells to perivascular tissues. In parallel, dysregulation of vasoactive mediators is characterised by increased endothelin-1 [31] production and reduced synthesis of nitric oxide and prostacyclin, contributing to vasoconstriction and impaired vascular homeostasis. These alterations are accompanied by progressive vascular remodelling, defective angiogenesis, and capillary rarefaction, which may contribute to chronic tissue hypoxia, oxidative stress, and increased production of reactive oxygen species, thereby further promoting inflammation and fibroblast activation [32]. Beyond classical vasoactive mediators, neuropeptide-mediated G protein-coupled receptor signalling may also contribute to cardiovascular homeostasis through the modulation of intracellular calcium dynamics and neuronal excitability [33].

Immune dysregulation represents an important component of the transition from vascular and tissue injury to fibrosis in SSc. Both innate and adaptive immune responses are involved early in the disease course, with perivascular inflammatory infiltrates comprising macrophages, dendritic cells, mast cells, T lymphocytes, and B lymphocytes. Among innate immune populations, alternatively activated M2 macrophages may contribute to fibrogenesis through the release of TGF-β, IL-6, CCL2, and other profibrotic mediators. In parallel, CD4+ T helper 2 (Th2) cells produce cytokines such as IL-4 and IL-13, which promote fibroblast activation, myofibroblast differentiation, and extracellular matrix (ECM) synthesis. B-cell dysregulation [34,35] may further participate in these processes through autoantibody production, cytokine secretion, and profibrotic signalling. The presence of B-cell infiltrates in lung tissue from patients with SSc-ILD provides additional evidence for their involvement in pulmonary immune–fibrotic responses, although these observations do not establish B cells as a primary driver of fibrosis. Innate immune pathways, including Toll-like receptor signalling and type I interferon responses, may further sustain immune activation and the production of inflammatory and profibrotic mediators [2,36,37].

Autoantibodies are detectable in the majority of patients with SSc and have important diagnostic, classification, and prognostic relevance. Among SSc-specific autoantibodies, anticentromere antibodies (ACA), anti-topoisomerase I antibodies (ATA; anti-Scl-70), and anti-RNA polymerase III antibodies (anti-RNAP III) are included in the 2013 American College of Rheumatology/European League Against Rheumatism (ACR/EULAR) classification criteria for SSc [3]. These autoantibodies are associated with distinct clinical phenotypes and patterns of organ involvement. ACAs are predominantly associated with lcSSc, whereas ATAs are more frequently associated with dcSSc and an increased risk of interstitial lung disease. Anti-RNAP III antibodies are predominantly associated with dcSSc and an increased risk of scleroderma renal crisis [38].

Beyond the autoantibodies incorporated into the classification criteria, other SSc-specific or SSc-associated autoantibodies, including anti-U3 RNP, anti-U1 RNP, anti-PM/Scl, anti-Ku, and anti-Th/To antibodies, define additional serological subsets with distinct clinical associations. From an SSc-ILD perspective, ATA are particularly relevant because of their well-established association with pulmonary involvement, whereas the clinical relevance of other autoantibody profiles varies according to the pulmonary outcome considered. Overall, the heterogeneity of SSc autoantibody profiles supports their role in clinical phenotyping and risk stratification, although their utility for detecting established ILD, predicting subsequent progression, or monitoring disease activity should be considered separately according to the available evidence [39].

Persistent fibroblast activation and myofibroblast accumulation are key features of SSc fibrosis and contribute to excessive extracellular matrix (ECM) deposition and progressive tissue remodelling. Multiple signalling pathways are involved in this process, with transforming growth factor-β (TGF-β) acting as an important mediator of fibroblast activation and profibrotic responses. Platelet-derived growth factor (PDGF) signalling may further contribute to fibroblast proliferation and ECM production. Activated fibroblasts and myofibroblasts subsequently produce increased amounts of ECM components, including collagen and fibronectin, thereby promoting progressive fibrotic remodelling [40,41,42].

Within the lung, vascular and epithelial injury, immune dysregulation, and fibroblast activation converge to establish a profibrotic microenvironment characterised by persistent fibroblast activation and excessive ECM deposition. Fibroblasts represent major effector cells in SSc fibrosis and exhibit functional alterations that may contribute to the persistence of the fibrotic response [41]. Sustained fibroblast activation can disrupt the balance between ECM synthesis and degradation, promoting progressive remodelling of pulmonary tissue and contributing to the development and progression of SSc-ILD [43].

**Figure 1:**
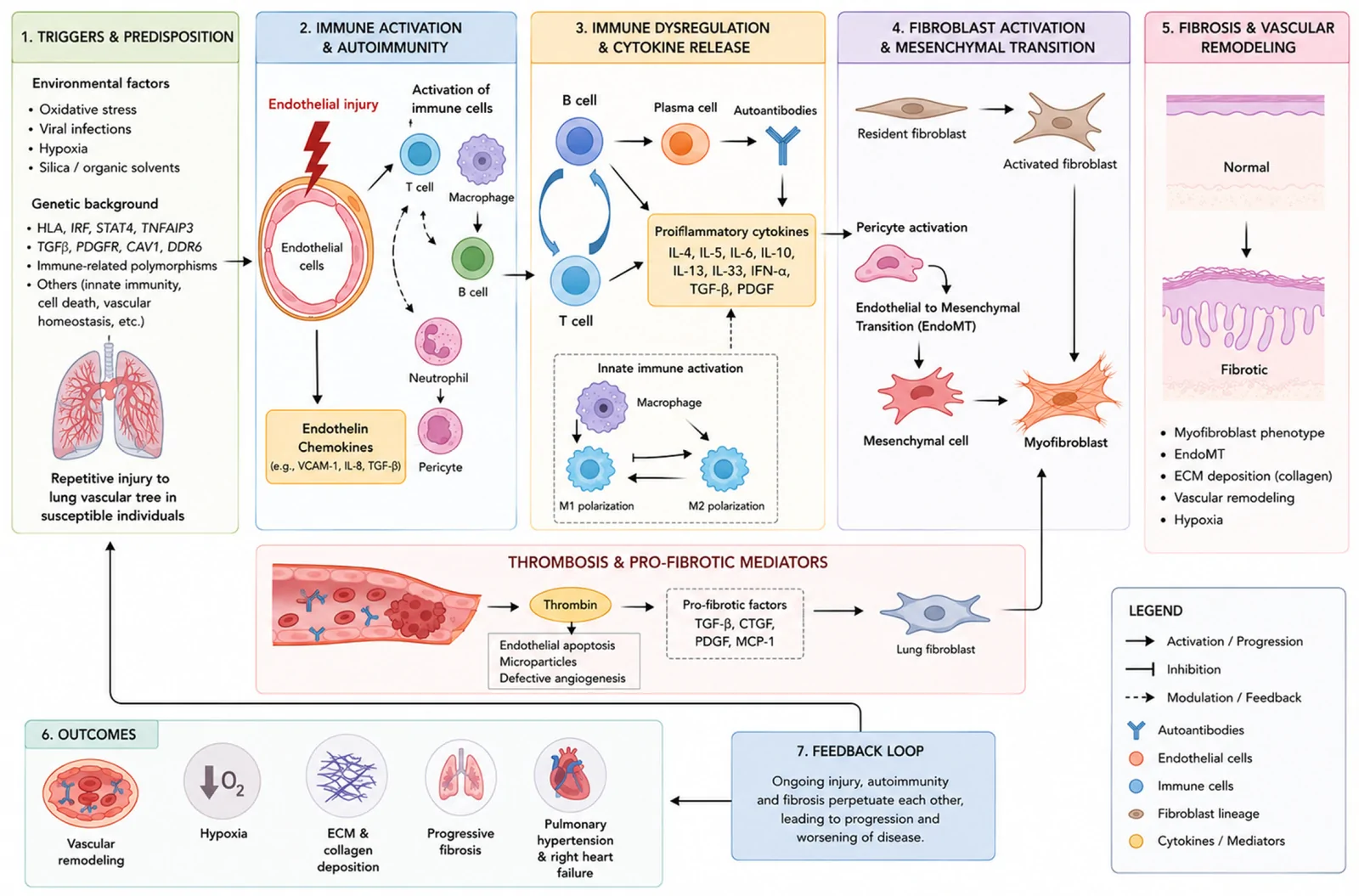
Pathogenesis of systemic sclerosis.

Collectively, SSc-ILD develops within a complex pathogenic network in which vascular and epithelial injury, immune dysregulation, and persistent fibroblast activation interact through overlapping pathways. Rather than being attributable to a single pathogenic mechanism, pulmonary fibrosis in SSc appears to result from the convergence of multiple interconnected processes. This framework provides a biological rationale for investigating biomarkers that reflect distinct components of SSc-ILD pathobiology and for evaluating their potential roles in risk stratification, disease monitoring, and assessment of therapeutic response [44] (Figure 1).

## 4. Biomarkers

### 4.1. Autoantibodies

Autoantibodies represent central biomarkers in systemic sclerosis, with key roles in diagnosis, disease classification, and prognostic stratification, detectable in over 95% of patients. Most patients are seropositive for antinuclear antibodies and develop disease-specific autoantibodies, often detected even prior to clinical onset, that define distinct clinical subsets and patterns of organ involvement. Among these, the most clinically relevant antibodies include anticentromere antibodies (ACA), anti-topoisomerase I antibodies (anti-Scl-70), and anti-RNA-polymerase III antibodies, which represents the principal serological subsets in systemic sclerosis and form the basis of ACR/EULAR classification criteria [45,46,47].

A large-scale registry-based analysis including 863 patients from the German Network for Systemic Scleroderma Registry demonstrated that a limited panel of five autoantibodies—ACA, ATA, anti-RNA polymerase III, anti-U1 RNP, and anti-PM-Scl—captures over 95% of systemic sclerosis-associated antibody responses in ANA-positive individuals. Antinuclear antibodies were detected in 94.2% of the cohort, while these disease-specific autoantibodies were largely mutually exclusive, with co-occurrence observed in only 1.6% of cases. Importantly, these autoantibody subsets were strongly associated with distinct clinical phenotypes, with ACA linked to limited cutaneous disease and vascular complications, and anti-topoisomerase I antibodies associated with diffuse disease and pulmonary fibrosis [48].

Given that pulmonary complications- specifically ILD and PAH- are the leading cause of mortality in SSc, identifying specific autoantibody profiles is mandatory for early diagnosis and specialised follow-up. According to the comprehensive evidence synthesised by Cavazzana et al., these biomarkers are not merely diagnostic adjuncts but are intrinsically linked to distinct pulmonary trajectories, allowing for the classification of patients into specific fibro-proliferative, vascular, or high-risk mixed phenotypes [49,50], (Table 1).

#### 4.1.1. The Fibro-Proliferative Phenotype: Anti-Topoisomerase I (ATA/Anti-Scl-70)

Anti-topoisomerase I antibodies (ATA; anti-Scl-70) are among the most clinically relevant serological markers associated with SSc-ILD. ATA positivity is more frequently observed in patients with diffuse cutaneous systemic sclerosis (dcSSc) and is associated with an increased risk of ILD and more extensive pulmonary involvement [51]. Longitudinal studies have further associated ATA positivity with a greater risk of pulmonary function deterioration, particularly during the early years of disease, when clinically relevant declines in forced vital capacity (FVC) and diffusing capacity of the lung for carbon monoxide (DLCO) may occur [52,53]. Accordingly, ATA status may contribute to pulmonary risk stratification and, together with clinical characteristics and pulmonary function parameters, help identify patients who warrant careful assessment for SSc-ILD [54]. ATA positivity has been consistently associated with an increased risk of clinically relevant ILD across SSc cutaneous subsets, although pulmonary involvement is more frequently encountered in patients with diffuse cutaneous disease. ATA-positive patients have also been reported to have less favourable survival, particularly in the context of dcSSc [55]. Beyond pulmonary involvement, ATA positivity has been associated with digital vascular and musculoskeletal manifestations, supporting its association with a broader clinical phenotype. Experimental and translational evidence further suggests that immune responses associated with topoisomerase I may participate in fibroblast activation and extracellular matrix remodelling; however, the extent to which these mechanisms independently contribute to progressive pulmonary fibrosis remains incompletely defined [56].

The prognostic implications of ATA positivity appear to depend partly on the clinical context and cutaneous phenotype. In a Japanese SSc cohort, Hamaguchi et al. reported pulmonary fibrosis in 84% of ATA-positive patients, compared with 7% of patients with ACA and 17% of those with anti-RNA polymerase antibodies [57]. ATA-positive patients also had a 10-year cumulative survival rate of approximately 70%, supporting an association between this serological profile, pulmonary involvement, and a less favourable disease course in this population [57]. However, these findings should not be interpreted as establishing ATA as an independent determinant of mortality, as prognosis may also be influenced by disease subtype, extent of organ involvement, and other clinical characteristics.

The relationship between ATA and outcome is further influenced by cutaneous phenotype. In a longitudinal cohort, ATA-positive patients with lcSSc exhibited a substantially higher prevalence of ILD (49%) than ATA-negative patients with lcSSc, approaching that observed in patients with diffuse cutaneous disease. Despite this increased burden of pulmonary involvement, cumulative survival in ATA-positive lcSSc was approximately 75% and was comparable to that of ATA-negative lcSSc, while remaining more favourable than in ATA-positive dcSSc. ATA-positive lcSSc patients were also more likely to develop diffuse cutaneous involvement during follow-up, with most transitions occurring within the first five years. Collectively, these observations suggest that ATA positivity identifies a subgroup at increased risk of ILD even in the presence of a limited cutaneous phenotype, whereas its prognostic implications for survival should be interpreted in conjunction with cutaneous subtype and overall disease phenotype [58].

#### 4.1.2. The Vascular-Predominant Phenotype: Anticentromere Antibodies (ACAs)

In contrast to the predominantly interstitial involvement associated with the fibro-proliferative phenotype, the ACA positivity is characterised by a relative sparing of the lung interstitium and a greater propensity for pulmonary microangiopathy. ACAs are predominantly associated with lcSSc, a lower risk of severe ILD [47,63], and a greater propensity for PAH, an important pulmonary complication of lcSSc [64].

The clinical significance of ACA, however, varies according to the pulmonary outcome considered. Although their presence may facilitate the identification of patients with a predominantly vascular pulmonary phenotype, their prognostic value after PAH has developed appears more limited. Data from the PHAROS registry showed no significant association between ACA positivity and overall survival, and ACA status was not identified as an independent predictor of mortality among patients with SSc-PAH [65]. Thus, ACA may be more informative for phenotypic characterization and risk stratification than for predicting clinical outcomes once PAH is established.

Nevertheless, the relationship between ACA and pulmonary involvement is more complex than a strictly lung-sparing profile. Caetano et al. identified a distinctive phenotype among ACA-positive patients with dcSSc, characterised by a more gradual evolution of cutaneous and internal organ involvement. ILD was more frequent in ACA-positive dcSSc than in ACA-positive lcSSc, but less frequent than in ACA-negative dcSSc. Moreover, ILD development extended over a longer disease course in the ACA-positive diffuse subset, whereas scleroderma renal crisis occurred less frequently and survival was more favourable than in ACA-negative dcSSc. Collectively, these findings suggest that ACA status may contribute to phenotypic stratification even within diffuse cutaneous disease and indicate that ACA positivity does not preclude clinically relevant interstitial lung involvement [63].

#### 4.1.3. The Multisystem Phenotype: Anti-RNA Polymerase III (Anti-RNAP III)

Anti-RNA polymerase III antibodies (anti-RNAP III) characterize a clinical phenotype distinct from that associated with ACA and are particularly linked to rapid disease onset and accelerated progression of skin thickening. Cavazzana et al. reported a shorter interval between the onset of Raynaud’s phenomenon and the first non-Raynaud manifestation in patients with isolated anti-RNAP III compared with those positive for anti-topoisomerase I antibodies, together with more rapid early skin thickening. However, rates of internal organ involvement and survival were similar between the two groups [66].

Beyond the cutaneous phenotype, anti-RNAP III positivity is strongly associated with scleroderma renal crisis [7,67]. Associations with malignancy have also been reported, particularly a close temporal relationship between cancer diagnosis and SSc onset in anti-RNAP III-positive patients [68,69,70]. These distinct systemic associations further support the consideration of anti-RNAP III and ACA as separate serological and clinical phenotypes.

From an SSc-ILD perspective, anti-RNAP III is less strongly associated with interstitial pulmonary involvement than anti-topoisomerase I antibodies (ATA). Severe interstitial fibrosis has been reported less frequently in anti-RNAP III-positive patients [7]; however, this serological profile should not be interpreted as excluding pulmonary parenchymal involvement. Current evidence does not establish anti-RNAP III as an independent predictor of either SSc-ILD development or subsequent progression.

Overall, anti-RNAP III appears to be more informative for clinical and serological phenotyping than as an established prognostic biomarker of SSc-ILD. Its presence identifies a distinct SSc subset characterised particularly by rapidly evolving cutaneous disease and an increased risk of scleroderma renal crisis, together with reported malignancy-related associations. Although pulmonary involvement may occur, available evidence does not currently support anti-RNAP III as an established biomarker of SSc-ILD progression [49].

#### 4.1.4. The Nucleolar and Overlap High-Risk Phenotype: Multi-Organ Vulnerability

The substantial burden of pulmonary involvement observed in patients harbouring antibodies that target nucleolar antigens or reflect overlap syndromes may include both ILD and PAH.

Anti-Th/To antibodies exemplify this profile: despite being associated with limited cutaneous disease, they are linked to a high prevalence of both ILD and PAH, affecting up to 75% of patients. This paradoxical combination of mild skin disease and severe pulmonary involvement has been associated with reduced survival compared to other lcSSc subsets, underscoring the importance of early detection [71].

Overlap-associated autoantibodies further expand this high-risk category. Anti-PM/Scl antibodies are typically associated with SSc–myositis overlap and a high prevalence of ILD; however, the pulmonary course is often more indolent and responsive to therapy, resulting in comparatively favourable outcomes [72,73]. By comparison, in a clinical cohort of 126 European Caucasian patients, the presence of ILD, confirmed by high-resolution computed tomography, was observed in 59% of anti-PM/Scl-positive individuals compared to 51% in antibody-negative patients, while reduced diffusion capacity was noted in 61% of cases. Despite these findings, no statistically significant association was identified between anti-PM/Scl positivity and either ILD occurrence or pulmonary function impairment, suggesting that anti-PM/Scl positivity may not independently identify an increased risk of ILD or pulmonary functional impairment in this cohort [74]. Similarly, anti-Ku antibodies are associated with an overlap phenotype characterised by myositis and ILD, with an intermediate clinical course in which both inflammatory and fibrotic features may be present [75,76]. A prevalence of ILD of approximately 71% has been reported among anti-Ku-positive SSc patients, markedly higher compared to the general SSc population. Radiographic evaluation most frequently demonstrates nonspecific interstitial pneumonia (NSIP) and usual interstitial pneumonia (UIP) patterns. Pulmonary involvement in this subset has been associated with an unfavourable prognosis [77].

Additional markers may further contribute to risk stratification within this group. Anti-U3 RNP (fibrillarin) antibodies are associated with diffuse disease, early onset, and increased risk of PAH and cardiac involvement, particularly in specific ethnic populations. Anti-Ro52 (TRIM21) antibodies, frequently coexisting with other autoantibodies, have emerged as potential markers of disease severity and have been independently associated with ILD progression, PAH, and increased mortality across multiple SSc subsets [78,79,80]. A recent large meta-analysis encompassing 11,751 patients, supported by additional cohort data, indicates that anti-TRIM21 (Ro52) antibodies are present in approximately one-quarter of systemic sclerosis cases. Although these antibodies commonly coexist with other SSc-specific autoantibodies, they have emerged as independent predictors of severe organ involvement. In particular, anti-TRIM21 seropositivity has been significantly associated with the presence of ILD [81]. In an exploratory study, Hamberg et al. (2023) demonstrated that anti-Ro52 antibodies are significantly enriched in the bronchoalveolar lavage (BAL) fluid of patients with new-onset SSc-ILD, often at ratios exceeding 50 times the levels found in serum, raising the possibility of a local pulmonary autoimmune process. Furthermore, longitudinal analysis showed an independent association between anti-Ro52 positivity and accelerated decline in lung function [82,83].

Recent advances have also identified novel autoantibodies that further define high-risk pulmonary phenotypes. Anti-U11/U12 RNP (RNPC3) antibodies have been associated with severe and rapidly progressive ILD, often accompanied by significant reductions in FVC and DLCO and reported associations with malignancy [45,84]. Similarly, anti-BICD2 and anti-eIF2B antibodies have been linked to ILD and overlap features, while anti-RuvBL1/2 antibodies define a multisystem phenotype with myositis, cardiac, and gastrointestinal involvement, occasionally accompanied by pulmonary disease [85].

Beyond classical serological markers, Chepy et al. (2025) highlight emerging antigenic targets, including anti-eIF2B, anti-RNPC3 (U11/U12), anti-BICD2, and telomere-associated antigens such as anti-TERF1, which may refine the classification of previously seronegative subsets and are associated with distinct clinical phenotypes, including severe interstitial lung disease and malignancy. These findings raise the possibility that some autoantibodies may participate in disease-related mechanisms rather than serving solely as diagnostic or prognostic markers. The occurrence of autoantibodies before clinical manifestations, together with reported associations between antibody titres and disease features, provides additional support for a potential contribution of humoral immune mechanisms to SSc pathogenesis. Clinical responses observed with B-cell-targeted therapies, including rituximab and, more recently, anti-CD19 CAR-T cell therapy, further support the involvement of B-cell-mediated immune mechanisms in SSc. Experimental models in which immunization with autoantigens such as topoisomerase I or PRMT5 induces SSc-like cutaneous and pulmonary fibrosis provide additional evidence for a potential contribution of humoral immune dysregulation to fibrotic processes. However, these observations do not establish humoral immunity as a primary driver of fibrosis, and its precise role in the initiation and progression of pulmonary fibrosis remains to be determined [86]. The anti-eIF2B is a rare autoantibody reported in approximately 2.5% of SSc cases. Its presence is strongly associated with the diffuse cutaneous variant of the disease and the development of ILD. Clinically, this specificity may be considered in SSc patients who test negative for traditional antinuclear antibodies (ANA) but display a distinct, high titre cytoplasmic staining pattern (≥1:320) during indirect immunofluorescence [87,88].

Anti-U1 RNA antibodies have been identified in approximately 61% of systemic sclerosis patients with anti-U1 RNP and have been associated with pulmonary involvement. Their presence has also been associated with a higher prevalence of pulmonary fibrosis and impaired gas transfer, suggesting potential value for ILD risk stratification [89].

Taken together, these findings underscore the clinical relevance of autoantibody-defined subsets in systemic sclerosis beyond conventional serological classification. Distinct autoantibody profiles characterise different patterns of ILD involvement and disease course, ranging from more indolent forms to progressive fibrotic phenotypes, and may therefore contribute to risk stratification and longitudinal assessment of SSc-ILD.

### 4.2. Serum Biomarkers for Interstitial Lung Disease in Systemic Sclerosis

The management of SSc-ILD is increasingly reliant on serum biomarkers that can provide dynamic assessment beyond the static measurements of pulmonary function tests. These molecules, reflecting various facets of alveolar injury and fibro-inflammatory signalling, are essential for identifying “progressors”—patients at high risk for significant decline in forced vital capacity (FVC) and diffusing capacity (DLCO) (Table 2).

#### 4.2.1. Markers of Alveolar Epithelial Injury and Type II Pneumocyte Activity

Krebs von den Lungen-6 (KL-6) and surfactant proteins, particularly surfactant protein D (SP-D), represent circulating biomarkers related to alveolar epithelial injury and type II pneumocyte activity. KL-6 is a high-molecular-weight MUC1 glycoprotein expressed by type II alveolar epithelial cells, with increased circulating concentrations reported in several forms of ILD, including SSc-ILD. Available evidence suggests that KL-6 may provide information on different aspects of pulmonary involvement; however, its association with the presence and extent of ILD should be distinguished from its potential prognostic value for subsequent disease progression [94].

From a cross-sectional perspective, elevated KL-6 concentrations have been associated with greater radiological disease extent and impaired pulmonary function. Sieiro Santos et al. found higher KL-6 concentrations in patients with SSc-ILD than in those without ILD, together with inverse correlations with FVC and DLCO. KL-6 levels were also associated with semiquantitative HRCT extent, with higher concentrations observed in patients with more extensive pulmonary involvement [95]. Consistent with these observations, Lee et al. reported higher serum KL-6 levels in patients with more severe connective tissue disease-associated ILD, further supporting an association between KL-6 and the overall burden of pulmonary involvement [96]. These findings support the potential utility of KL-6 for characterizing the presence and baseline severity of ILD; however, the cross-sectional nature of the available evidence precludes conclusions regarding its prognostic value for subsequent pulmonary deterioration [95,96].

Longitudinal data suggest that KL-6 may provide prognostic information in early SSc-ILD. In the prospective GENISOS cohort, Salazar et al. found that higher baseline KL-6 levels were associated with greater FVC decline over one year, with concentrations above 1273 U/mL corresponding to an approximately 7% greater annualized decline. In contrast, baseline CCL18 was not associated with short-term FVC deterioration, differing from findings from Scleroderma Lung Study II, where higher CCL18 levels were associated with subsequent FVC and DLCO decline. These discrepancies may reflect differences in patient characteristics, disease severity, treatment exposure, follow-up duration, or definitions of progression. Collectively, these findings suggest a potential role for KL-6 in identifying patients at risk of early functional decline, although its prognostic value and proposed thresholds require further evaluation in independent SSc-ILD cohorts [92].

SP-D is another circulating marker of alveolar epithelial and alveolar–capillary injury that has been investigated in SSc-ILD [97]. Elevated serum SP-D concentrations have been associated with pulmonary involvement and greater ILD severity. Hant et al. reported that both SP-D and KL-6 levels correlated with HRCT fibrosis scores, whereas neither marker was significantly associated with the extent of ground-glass opacity [98]. These findings suggest that their relationship with radiological abnormalities may differ according to the imaging features assessed and should not be interpreted as uniformly reflecting all components of ILD severity.

Comparative and longitudinal studies have further explored the potential clinical relevance of SP-D and KL-6. Yanaba et al. reported higher sensitivity for SP-D and greater specificity for KL-6 in detecting pulmonary fibrosis, while serial SP-D measurements appeared to reflect temporal changes in pulmonary involvement [99]. Similarly, Asano et al. found that elevated SP-D levels were associated with pulmonary fibrosis and impaired vital capacity and DLCO [100].

In a retrospective cohort of patients with SSc- or mixed connective tissue disease-associated ILD, Yamakawa et al. observed that longitudinal changes in KL-6 were associated with changes in FVC, whereas higher SP-D levels were associated with subsequent short-term FVC decline. Collectively, these observations suggest that KL-6 and SP-D may provide complementary information regarding longitudinal pulmonary assessment and risk of functional deterioration. However, the limited sample size, retrospective design, and inclusion of a mixed disease population in the latter study warrant caution when extrapolating these findings specifically to SSc-ILD [101].

IL-18 has also been evaluated as a circulating biomarker of pulmonary involvement in SSc. In the cross-sectional study by Sieiro Santos et al., serum IL-18 and KL-6 levels were higher in patients with SSc-ILD than in those without ILD and were inversely associated with FVC and DLCO. Both biomarkers were related to semiquantitative HRCT severity, although the association with extensive lung involvement was observed for KL-6 but not for IL-18. These findings suggest that IL-18 and KL-6 may provide complementary information regarding the presence and baseline severity of SSc-ILD. However, the cross-sectional design precludes conclusions regarding their ability to predict subsequent disease progression, and current evidence does not support their use as alternatives to longitudinal assessment by pulmonary function testing or HRCT [95].

Overall, KL-6 and SP-D are among the most extensively studied circulating markers of alveolar epithelial injury in SSc-ILD. Available evidence indicates associations with the presence and severity of pulmonary involvement, while longitudinal studies suggest that serial or baseline measurements may provide additional information regarding disease course and subsequent functional deterioration [92,94,95,96,97,98,99,101]. However, heterogeneity in study design, patient characteristics, disease stage, treatment exposure, biomarker thresholds, follow-up duration, and definitions of progression limits the comparability and generalizability of these findings. Accordingly, KL-6 and SP-D may currently be considered complementary to established clinical assessment, pulmonary function testing, and HRCT, whereas their independent and incremental value for monitoring and prognostic stratification requires further prospective evaluation.

#### 4.2.2. Chemokines and Macrophage-Driven Fibrogenesis

Chemokines involved in macrophage activation, leukocyte recruitment, and immune–fibrotic signalling have been investigated as circulating biomarkers in SSc-ILD. Among these, CCL18 has been associated with pulmonary fibrotic involvement and impaired lung function. Increased CCL18 concentrations have been reported in serum and bronchoalveolar lavage fluid, with several studies showing inverse associations with FVC and DLCO [102,103,104]. Longitudinal studies have further associated higher baseline CCL18 levels with subsequent deterioration in pulmonary function, progression of lung fibrosis, and reduced survival [103,104]. However, these findings have not been consistent across cohorts. In the prospective GENISOS cohort, Salazar et al. found no significant association between baseline CCL18 and short-term FVC decline in patients with SSc-ILD [92]. Differences in cohort characteristics, disease stage and severity, treatment exposure, follow-up duration, and definitions of progression may contribute to these discrepancies. Thus, CCL18 may provide prognostic information in selected populations, but current evidence does not support its uniform application as an independent predictor of SSc-ILD progression [103,104,105].

CCL2, also known as monocyte chemoattractant protein-1 (MCP-1), has been investigated as a marker of disease activity and subsequent pulmonary deterioration. Hasegawa et al. reported associations between circulating CCL2 levels and clinical disease activity, with longitudinal changes potentially reflecting changes in pulmonary and extrapulmonary manifestations [106]. More specifically, Wu et al. evaluated CCL2 in two independent cohorts of patients with early SSc and found that higher baseline concentrations were associated with greater subsequent FVC decline and increased mortality, with these associations persisting after adjustment for relevant clinical variables. These findings support the potential prognostic relevance of CCL2; however, its incremental value beyond established clinical, functional, and imaging predictors remains to be determined before routine application in SSc-ILD risk stratification [107]. CXCL4, predominantly released by plasmacytoid dendritic cells in SSc, has also been investigated in relation to pulmonary involvement. Van Bon et al. reported higher circulating CXCL4 levels in patients with more severe skin and lung fibrosis, while elevated baseline concentrations were associated with an earlier occurrence of pulmonary fibrosis. These observations suggest that CXCL4 may contribute to the identification of patients at increased risk of pulmonary involvement; however, further longitudinal studies are required to establish its independent and incremental prognostic value specifically in SSc-ILD [108].

CXCL10 has primarily been investigated as a marker of inflammatory activity in SSc. Higher circulating concentrations have been reported in patients with pulmonary and other organ involvement [109], while longitudinal changes in CXCL10 and CCL2 have been proposed to reflect changes in the inflammatory and fibrotic milieu during disease evolution [110]. In contrast, Hasegawa et al. did not identify a significant association between serum CXCL10 levels and the severity of pulmonary involvement. These inconsistent findings may reflect differences in disease stage, clinical phenotype, sample size, treatment exposure, and the pulmonary outcomes assessed. Accordingly, the current evidence is insufficient to define a specific role for CXCL10 in assessing SSc-ILD severity or predicting subsequent progression [106,109,110].

CX3CL1 (fractalkine) has also been investigated as a potential biomarker of pulmonary involvement in SSc. Increased circulating and tissue expression of CX3CL1 has been reported in SSc, with higher concentrations associated with the presence of ILD, greater radiological fibrotic burden, and impaired pulmonary function. Longitudinal observations further suggest an association between elevated CX3CL1 levels and subsequent ILD progression [111,112,113]. Collectively, these findings indicate that CX3CL1 may provide complementary information regarding pulmonary involvement and the risk of disease progression; however, its independent and incremental prognostic value beyond established clinical, functional, and imaging parameters remains to be determined.

#### 4.2.3. Pro-Inflammatory Cytokines and Extracellular Matrix Mediators

Beyond epithelial and macrophage markers, systemic inflammatory mediators such as Interleukin-6 (IL-6) and YKL-40 (Chitinase-3-like protein 1) provide crucial prognostic insights. IL-6 is a pleiotropic cytokine that drives both myofibroblast differentiation and systemic inflammation; elevated serum IL-6 has been robustly associated with worse pulmonary outcomes and increased mortality in SSc cohorts [114]. Interleukin-6 (IL-6) has been identified as a key mediator in the pathogenesis of systemic sclerosis, contributing to a self-sustaining cycle of inflammation, vascular injury, and fibrosis. Elevated serum IL-6 levels have been consistently observed in patients with systemic sclerosis-associated interstitial lung disease and are closely associated with early diffuse disease, increased skin thickening, heightened inflammatory activity, and greater overall disease severity, while also correlating with the extent and progression of both cutaneous and pulmonary involvement [45]. Moreover, increased IL-6 concentrations have been linked to impaired pulmonary function, highlighting its relevance as both a biomarker of disease activity and a promising therapeutic target in systemic sclerosis [115,116,117]. De Lauretis et al. demonstrated that serum interleukin 6 (IL-6) serves as a potent non-invasive biomarker for predicting early functional decline and mortality in patients with SSc-ILD. In an exploratory analysis of eight cytokines, IL-6 emerged as the only independent predictor of lung function deterioration, specifically regarding DLCO decline, in both SSc-ILD and idiopathic pulmonary fibrosis (IPF) cohorts [118]. While therapeutic IL-6 blockade has shown modest effects on skin involvement, its beneficial impact on preserving lung function highlights its potential as a targeted strategy for limiting interstitial lung disease progression in systemic sclerosis [115,119]. Furthermore, YKL-40, a glycoprotein involved in tissue remodelling and inflammation, serves as a significant marker for advanced ILD and poor survival. Clinically, elevated serum YKL-40 levels have been reported in patients with systemic sclerosis compared to healthy controls, with significantly higher concentrations observed in those with SSc-associated interstitial lung disease. A prospective study involving 88 patients identified a serum threshold above 275 mg/L as being associated with more severe pulmonary involvement and increased mortality, suggesting its potential value as a biomarker of disease severity and prognosis [120].

#### 4.2.4. MMPs (Matrix Metalloproteinases)

In systemic sclerosis-associated interstitial lung disease, chronic pulmonary injury promotes excessive collagen deposition and progressive disruption of normal lung architecture through complex interactions between structural and immune cells. Beyond extracellular matrix degradation, matrix metalloproteinases act as important regulators of fibrotic remodelling by modulating cytokine and growth factor activation [90,121].

Circulating MMP-7 (matrilysin) levels have been shown to be elevated in systemic sclerosis, supporting its involvement in fibrotic tissue remodelling. Increased concentrations are associated with pulmonary involvement, including lung fibrosis, dyspnoea, and impaired gas exchange, as well as with the diffuse disease subset and male sex, suggesting a link with more severe disease phenotypes. Although higher levels have also been observed in patients with pulmonary arterial hypertension, this association has not been consistently significant [122,123]. Increased MMP-9 expression appears particularly pronounced in diffuse cutaneous disease and correlates with the extent of skin fibrosis, as reflected by skin thickness scores, as well as with profibrotic signalling pathways, including transforming growth factor-β activity [124]. Manetti et al. (2012) identified matrix metalloproteinase-12 (MMP-12) as a key biomarker reflecting disease severity in SSc. The study found significantly elevated serum MMP-12 levels in SSc patients, which correlated positively with the extent of skin fibrosis (mRSS) and inversely with pulmonary function (%FVC) [125].

#### 4.2.5. Other Molecules

According to the multicentre cohort study conducted by the Canadian Scleroderma Research Group (CSRG), elevated C-reactive protein (CRP) levels were present in approximately 25.7% of patients with SSc. The research demonstrated that CRP elevation is significantly more prevalent in the dcSSc subset, particularly during the early stages of the disease (≤3 years from the first non-Raynaud’s symptom). Furthermore, the study identified that elevated CRP serves as a critical biomarker for higher disease activity, severity, and poor prognosis, showing significant associations with the modified Rodnan skin score (MRSS), reduced pulmonary function (TLC and FVC < 80% predicted), and increased serum creatinine [126,127].

### 4.3. Immunogenetic and Cellular Biomarkers in SSc-ILD: From Susceptibility to Pulmonary Progression

The immunogenetic background of SSc contributes to disease susceptibility and to the development of distinct clinical and serological phenotypes. Associations involving human leukocyte antigen (HLA) loci have been identified across different SSc subsets, with particularly relevant differences according to autoantibody status. Specific HLA class II variants have been associated with anticentromere antibody (ACA) and anti-topoisomerase I antibody (ATA) positivity, supporting a relationship between genetic background and serological heterogeneity in SSc. Given the markedly different patterns of pulmonary involvement associated with these autoantibody profiles, such immunogenetic associations may contribute indirectly to the heterogeneity observed in SSc-ILD. However, current evidence primarily supports their relevance to SSc susceptibility and serological stratification rather than their use as independent biomarkers for ILD detection or prediction of pulmonary disease progression [128,129].

Additional immunogenetic complexity is suggested by associations involving HLA class I alleles and killer cell immunoglobulin-like receptor (KIR) loci. Hanson et al. reported an association of the HLA-B44:03–HLA-C16:01 haplotype with SSc and described interactions between KIR and HLA class I loci that may contribute to the clinical and immunological heterogeneity of the disease. However, the specific relevance of these immunogenetic associations to pulmonary fibrosis remains unclear, and their potential utility for SSc-ILD risk stratification requires further investigation [130].

More direct evidence for a cellular biomarker relevant to pulmonary involvement was provided by Fava et al. in patients with SSc positive for anti-topoisomerase I antibodies. Circulating topoisomerase I-specific CD4+ T cells exhibited a predominantly Th17-associated phenotype, with higher frequencies observed in patients with ILD. Moreover, increased levels of these autoreactive T cells were related to subsequent deterioration in pulmonary function, including FVC decline, in both retrospective and prospective analyses. These findings suggest that this cellular marker may have potential value for identifying pulmonary involvement and stratifying the risk of subsequent functional deterioration. However, confirmation in larger, independent cohorts is required before topoisomerase I-specific CD4+ T-cell frequency can be considered suitable for clinical risk stratification or longitudinal monitoring in SSc-ILD [131].

Overall, available immunogenetic studies provide insight into the biological heterogeneity of SSc but currently have limited direct applicability to SSc-ILD assessment. HLA and KIR associations appear to be more informative for disease susceptibility and serological or clinical phenotyping, whereas circulating topoisomerase I-specific CD4+ T cells have shown a more direct relationship with both ILD presence and subsequent pulmonary functional decline. Further longitudinal studies are needed to determine whether these emerging cellular and immunogenetic biomarkers provide incremental clinical value beyond established autoantibody profiles, pulmonary function testing, and HRCT-based assessment [128,129,130,131].

## 5. Biomarkers in Therapeutic Stratification and Response Assessment in SSc-ILD

The expanding therapeutic landscape of SSc-ILD has increased interest in biomarkers that may complement clinical assessment, pulmonary function testing (PFT), and high-resolution computed tomography (HRCT) in evaluating treatment response. Importantly, biomarkers associated with disease severity or spontaneous progression should be distinguished from predictive biomarkers that identify differential responses to specific therapies and from monitoring biomarkers that reflect changes during treatment. Although several circulating, serological, and imaging markers have shown potential in these settings, evidence supporting biomarker-guided therapeutic selection in SSc-ILD remains limited, and most candidates require prospective validation before routine clinical implementation [132].

Evidence from the Scleroderma Lung Studies provides a clinical framework for evaluating potential markers of treatment response. Scleroderma Lung Study I (SLS I) demonstrated a modest beneficial effect of oral cyclophosphamide on pulmonary function in symptomatic SSc-ILD, although the treatment effect was largely no longer apparent one year after therapy was discontinued [133,134]. In Scleroderma Lung Study II (SLS II), mycophenolate mofetil (MMF) and cyclophosphamide were associated with comparable improvements in FVC over 24 months, with MMF showing a more favourable tolerability profile [135]. Quantitative HRCT analyses subsequently demonstrated reductions in radiological ILD burden following immunosuppressive therapy, supporting imaging-derived measures as potential tools for longitudinal assessment of treatment-associated changes rather than as established predictors of therapeutic response [136].

Circulating biomarkers of epithelial injury and inflammation have also been investigated in relation to cyclophosphamide response. Sumida et al. reported that an unfavourable response to intravenous cyclophosphamide was associated with lower pretreatment DLCO and higher baseline concentrations of KL-6, SP-D, CRP. During treatment, persistently elevated KL-6 concentrations were more frequently observed among poor responders, whereas declining SP-D levels were associated with a more favourable therapeutic course. These findings suggest that serial KL-6 and SP-D measurements may provide complementary information for monitoring treatment response. However, given the observational design and limited sample size, the proposed biomarker thresholds should be regarded as exploratory rather than established criteria for therapeutic decision-making [137].

The IL-6 pathway provides another example of the potential relationship between inflammatory phenotype and therapeutic response. In the phase II faSScinate and phase III focuSSced trials, tocilizumab did not meet the primary efficacy endpoints related to cutaneous disease, although preservation of FVC was observed as a secondary pulmonary outcome [138,139]. Subsequent analysis of the focuSSced SSc-ILD population by Ghuman et al. specifically distinguished prognostic factors from potential treatment-predictive marker. Higher baseline IL-6 concentrations were associated with greater subsequent FVC decline in placebo-treated patients, while several clinical and biological variables were investigated in relation to the pulmonary treatment effect of tocilizumab. These observations illustrate the potential value of integrating inflammatory and serological profiles into treatment-response models; however, they do not yet define a biomarker-selected subgroup in which response to tocilizumab can be reliably predicted [140].

B-cell-directed therapy represents another setting in which biomarker-based stratification could potentially refine therapeutic selection. Observational studies have reported stabilization or improvement of pulmonary function following rituximab treatment in some patients with SSc-ILD [141,142,143,144]. These observations support further investigation of B-cell-associated pathways but should not be interpreted as confirming humoral immunity as a primary driver of pulmonary fibrosis. Moreover, no circulating or cellular B-cell biomarker has yet been established for identifying patients with SSc-ILD who are more likely to respond to rituximab. The EVER-ILD trial reported greater improvement in FVC with rituximab combined with MMF than with MMF alone in patients with an NSIP pattern of ILD [145]; however, the heterogeneous underlying diagnoses limit direct extrapolation of these findings specifically to SSc-ILD.

The final RECITAL trial compared rituximab with intravenous cyclophosphamide in patients with severe or progressive connective tissue disease-associated ILD, including SSc. Both treatment groups showed improvement in FVC, but rituximab was not superior to cyclophosphamide for the primary FVC endpoint at 24 weeks. Because RECITAL included patients with SSc, idiopathic inflammatory myositis, and mixed connective tissue disease and did not establish a biomarker capable of predicting differential treatment response, its relevance to biomarker-guided management lies primarily in highlighting the need for markers that can identify patients more likely to benefit from alternative immunomodulatory strategies [146].

Evidence linking biomarkers to antifibrotic treatment response in SSc-ILD remains comparatively limited. The LOTUSS study primarily evaluated the safety and tolerability of pirfenidone in SSc-ILD. More recently, Scleroderma Lung Study III evaluated MMF plus pirfenidone versus MMF plus placebo. No statistically significant difference in the primary FVC endpoint was observed between the treatment groups, although interpretation was limited by substantially lower enrolment than planned and consequent loss of statistical power. Accordingly, current evidence does not establish a circulating biomarker capable of predicting individual response to pirfenidone in SSc-ILD [147,148].

For nintedanib, the SENSCIS trial demonstrated a reduction in the annual rate of FVC decline in patients with SSc-ILD [149], while INBUILD provided evidence for nintedanib across a broader population with progressive fibrosing ILDs [150,151]. However, therapeutic efficacy at the population level should not be interpreted as evidence of an individual treatment-predictive biomarker. Although circulating biomarkers are increasingly being investigated in progressive pulmonary fibrosis, heterogeneity in underlying ILD diagnoses, disease stage, and treatment exposure limits direct extrapolation to SSc-ILD. Identification of biomarkers capable of predicting or dynamically monitoring antifibrotic response therefore remains an important area for prospective investigation.

Emerging molecular approaches may provide additional opportunities to integrate mechanistic biomarkers with targeted treatment. Experimental studies suggest that Janus kinase (JAK) inhibition can modulate inflammatory and profibrotic macrophage pathways relevant to SSc-ILD, while a phase I/II study of tofacitinib demonstrated modulation of interferon-regulated molecular signatures in skin from patients with early diffuse cutaneous SSc. However, these observations neither establish therapeutic efficacy in SSc-ILD nor validate these molecular signatures as pulmonary treatment-response biomarkers. Their relevance to SSc-ILD should therefore be considered mechanistic and hypothesis-generating pending studies incorporating pulmonary-specific molecular and clinical endpoints [152,153].

Overall, current evidence supports a distinction between biomarkers of disease prognosis, biomarkers reflecting longitudinal treatment-associated changes, and biomarkers capable of predicting differential therapeutic response. Serial KL-6 and SP-D measurements during cyclophosphamide therapy and inflammatory or serological profiles evaluated in tocilizumab-treated populations provide preliminary examples of treatment-related biomarker applications, but none currently has sufficient evidence to guide routine therapeutic selection. Future prospective studies should determine whether circulating, cellular, imaging, or composite biomarkers provide incremental information beyond established clinical characteristics, PFT, and HRCT and, critically, whether biomarker-defined subgroups derive differential benefit from specific immunomodulatory or antifibrotic therapies.

## 6. Conclusions

The landscape of Systemic Sclerosis-associated Interstitial Lung Disease (SSc-ILD) is being fundamentally reshaped by the emergence of high-precision biomarkers. This review highlights that while autoantibody profiling—specifically the presence of anti-topoisomerase I (Scl-70)—remains a cornerstone for initial risk stratification, it is the integration of dynamic biochemical markers that offers the most promising avenue for real-time monitoring.

Epithelial cell-derived proteins, particularly KL-6 and SP-D, have emerged as candidate circulating biomarkers of alveolar epithelial injury and pulmonary fibrosis, with longitudinal studies suggesting potential value for monitoring disease course and stratifying the risk of functional deterioration. In parallel, inflammatory mediators, including IL-6 and CCL18, reflect components of immune activation and profibrotic signalling and may provide complementary information regarding SSc-ILD activity and progression.

Despite these advancements, the transition from “candidate markers” to “validated clinical endpoints” remains incomplete. The next frontier in SSc-ILD research must involve the development of multi-parametric biosignatures that synthesize serological data with genomic and radiomic features. Moving forward, large-scale longitudinal validation within standardized international cohorts is mandatory to establish robust cut-off values. Ultimately, leveraging these molecular insights will be instrumental in achieving the goal of personalized therapeutic stewardship, allowing for the timely initiation of antifibrotic or targeted immunosuppressive regimens before irreversible structural lung damage occurs.

## Figures and Tables

**Table 1:** Autoantibodies in Systemic Sclerosis.

Autoantibody		Antigen Target	Cutaneous Phenotype	Pulmonary Involvement	Prognostic Implications
Autoantibodies included in the 2013 ACR/EULAR classification criteria	Anti-Topoisomerase I (ATA/Scl-70) [45,46,47,48,49,51,52,53,54,55,56,57,58,59,60,61,62]	DNA Topoisomerase I	Diffuse	Severe, progressive ILD; early FVC/DLCO decline	Poor prognosis; high mortality
	Anti-RNA polymerase III (anti-RNAP III) [46,47,48,49,59,60,61,62]	RNA Polymerase III	Rapid diffuse	Moderate ILD (~18%); possible PAH	Aggressive early disease
	ACA (anti-centromere) [45,46,47,48,49,59,61,62]	Centromere proteins	Limited	Low ILD risk; high PAH risk (10–20%), ↓DLCO	Better survival; PAH main mortality driver
Other SSc-specific or SSc-associated autoantibodies	Anti-Th/To [47]	RNase MRP complex	Limited	High ILD + PAH (~70–75%)	Reduced survival
	Anti-U3 RNP [47]	Fibrillarin	Diffuse/mixed	Increased PAH risk; variable ILD	Severe phenotype
	Anti-RNPC3 [46,48,49]	U11/U12 RNP	Mixed	Severe ILD	High-risk subset
	Anti-U1 RNP [45,47,48,49,60]	U1 RNP complex	Overlap	ILD and PAH	Overlap phenotype
	Anti-PM/Scl [45,47,48,49,60]	PM/Scl complex	Overlap	ILD (often slower progression)	More favourable pulmonary outcome
	Anti-Ku [45,47,48,49]	Ku protein	Overlap	ILD	Intermediate prognosis
	Anti-Ro52 [45,48,49]	TRIM21	Overlap/mixed	ILD and PAH	Increased mortality risk
	Anti-NOR90 [49]	NOR90	Limited	Possible ILD	Generally favourable
	ANCA [46]	Neutrophil cytoplasm	Overlap	ILD, vasculitis lung involvement	Higher mortality
	Anti-RNA Pol I [46,48]	RPA194	Mixed	Moderate involvement	Possibly lower cancer risk

**Table 2:** Clinical roles and potential utility of circulating biomarkers in systemic sclerosis-associated interstitial lung disease (SSc-ILD).

Biomarker	Pathophysiological Mechanism	Early Detection and Baseline Severity	Prediction of Disease Progression and Prognosis	Disease Activity Monitoring	Treatment Response/Predictive Utility
KL-6 [9,59,61,62,90,91,92]	Type II pneumocyte proliferation and basement membrane disruption	Associated with greater HRCT extent; Inversely correlated with baseline FVC and DLCO Correlates with initial ground-glass opacities	Higher levels have been associated with subsequent functional decline and adverse pulmonary outcomes. KL-6 has shown comparatively consistent evidence for prediction of progression	Serial changes in KL-6 may provide information on disease evolution; increasing levels have been associated with subsequent ILD progression	Changes in KL-6 levels during treatment may reflect variations in disease activity; however, its utility for predicting treatment response has not been established
CCL18 [59,60,61,90,91,92]	M2 Macrophage Activation	Associated with greater fibrotic burden and pulmonary involvement	Associated with functional deterioration and adverse outcomes, although findings on short-term progression are inconsistent	Persistent or changing CCL18 levels may reflect ongoing profibrotic activity associated with macrophage activation	Changes in CCL18 levels during treatment have been reported; however, their utility for predicting treatment response remains unestablished
SP-D [59,60,61,62,90,91]	Alveolar-capillary barrier disruption; leakage into systemic circulation	Associated with pulmonary involvement and ground-glass abnormalities	Elevated baseline levels have been associated with subsequent functional deterioration in some cohorts	Longitudinal changes in SP-D levels may reflect variations in alveolar epithelial injury and pulmonary involvement	The utility of SP-D for predicting treatment response has not been established
IL-6 [9,59,60,61,90]	Pro-inflammatory signalling; acute-phase response and systemic immune activation	Associated with early active disease and greater disease severity	Higher baseline levels have been associated with early FVC decline and adverse outcomes	Longitudinal changes in IL-6 levels may reflect variations in systemic inflammatory activity	The role of IL-6 in predicting therapeutic response remains to be determined
CXCL4 [9,60,61,91]	Plasmacytoid dendritic cell activation	Biomarker of early systemic disease and pulmonary involvement	Higher levels have been associated with disease severity and progression in some cohorts	Changes in CXCL4 levels may reflect variations in immune activation, but their value for longitudinal disease monitoring remains uncertain	The role of CXCL4 in predicting treatment response remains unclear
YKL-40 [61,90]	Extracellular matrix remodelling, macrophage activation, and tissue repair dysregulation	Associated with established fibrosis and structural lung abnormalities	Higher levels have been associated with adverse outcomes	Variations in YKL-40 levels may reflect changes in extracellular matrix remodelling, although their value for disease monitoring remains uncertain	The predictive value of YKL-40 for treatment response remains uncertain
CRP [45,59,61,90,93]	Systemic inflammation- Acute-phase reactant	Associated with systemic inflammation and pulmonary involvement, but lacks specificity for SSc-ILD	Higher levels have been associated with functional decline and adverse outcomes in some cohorts	Changes in CRP may reflect systemic inflammation, although their value for monitoring SSc-ILD activity remains limited	The role of CRP in predicting treatment response in SSc-ILD remains unclear
CCL-2 [9,59,60,61,90,91]	Fibroblast stimulation, myofibroblasts differentiation, lymphocyte T trafficking and lymphocyte Th2 phenotype polarisation	Associated with pulmonary involvement and impaired lung function	Associated with progressive functional impairment	Changes in CCL2 levels may reflect variations in inflammatory cell recruitment and profibrotic activity	The role of CCL2 in predicting treatment response remains uncertain
CXCL10 [60,61,90]	Promoting leukocyte recruitment-chemoattractant for Th1 lymphocytes	Associated with inflammatory activity in early SSc	Associated with subsequent pulmonary functional deterioration	Changes in CXCL10 levels may reflect variations in inflammatory immune activity, although their value for longitudinal disease monitoring remains uncertain	Whether CXCL10 can predict treatment response requires further investigation
MMP-7 MMP-9 MMP-12 [60,61,90,91]	Extracellular matrix remodelling	Associated with pulmonary fibrosis and disease severity	MMP-7 has been associated with impaired lung function and progression; evidence for MMP-9/MMP-12 is less established	Changes in circulating MMP levels may reflect ongoing extracellular matrix turnover and remodelling, although their value for longitudinal disease monitoring remains uncertain	Their potential role in predicting treatment response remains to be determined

## Data Availability

The original contributions presented in this study are included in the article. Further inquiries can be directed to the corresponding author.

## Abbreviations

The following abbreviations are used in this manuscript:

SSc	Systemic Sclerosis
SSc-ILD	Systemic sclerosis- associated interstitial lung disease
ILD	Interstitial Lung Disease
dcSSc	diffuse cutaneous systemic sclerosis
lcSSc	Limited cutaneous systemic sclerosis
IL-6	Interleukin-6
KL-6	Krebs von den Lungen-6
HRCT	High resolution computed tomography
FVC	Forced vital capacity
PAH	Pulmonary arterial hypertension
SP-D	Surfactant protein D
CCL18	C-C motif chemokine ligand 18
CCL2	C-C motif chemokine ligand 2
CXCL4	Platelet Factor 4 or PF4
YKL-40	Chitinase-3-like protein 1
CXCL8	Interleukin-8
CX3CL1	Fractalkine
MMF	Mycophenolate mofetil
NSIP	Nonspecific interstitial pneumonia
UIP	Usual interstitial pneumonia
IPF	Idiopathic pulmonary fibrosis
MRSS	Modified Rodnan skin score
DLCO	Diffusion Capacity for Carbon Monoxide
PDGF	platelet-derived growth factor
TGF-β	transforming growth factor-β
CTGF	connective tissue growth factor
VEGF	vascular endothelial growth factor
CXCL10	Interferon gamma-induced protein 10
JAK	Janus kinase
CYC	cyclophosphamide
MMP	Matrix metalloproteinases
STAT	Signal Transducer and Activator of Transcription
